# Is antenatal care preparing mothers to care for their newborns? A community-based cross-sectional study among lactating women in Masindi, Uganda

**DOI:** 10.1186/1471-2393-14-114

**Published:** 2014-03-25

**Authors:** Richard Mangwi Ayiasi, Simon Kasasa, Bart Criel, Christopher Garimoi Orach, Patrick Kolsteren

**Affiliations:** 1 Makerere University School of Public Health, College of Health Sciences, Kampala Uganda, P. O. Box 7072, Kampala, Uganda; 2 Institute of Tropical Medicine, Antwerp Nationalestraat 155, B-2000 Antwerp, Belgium

## Abstract

**Background:**

Neonatal mortality has remained resistant to change in the wake of declining child mortality. Suboptimal newborn care practices are predisposing factors to neonatal mortality. Adherence to four ANC consultations is associated with improved newborn care practices. There is limited documentation of this evidence in sub-Saharan Africa where suboptimal newborn care practices has been widely reported.

**Methods:**

Structured interviews were held with 928 women having children under-five months old at their homes in Masindi, Uganda, from October-December 2011. Four/more ANC consultations (sufficient ANC) was considered the exposure variable. Three composite variables (complete cord care, complete thermal care and complete newborn vaccination status) were derived by combining related practices from a list of recommended newborn care practices. Logistic regression models were used to assess for associations.

**Results:**

One in five women 220(23.7%) were assessed to practice complete cord care. Less than ten percent 57(6.1%) were considered to practice complete thermal care and 611(65.8%) were assessed to have complete newborn vaccination status. Application of substance on the cord 744 (71.6%) and early bathing 816 (87.9%) were main drivers of sub-optimal newborn care practices. Multivariable logistic models did not demonstrate significant association between four/more ANC consultations and complete cord care, complete thermal care or complete newborn vaccination status. Secondary or higher education was associated with complete cord care [adjusted Odds Ratio (aOR): 2.72; 95% CI: 1.63-4.54] and complete newborn vaccination [aOR: 1.37; 95% CI: 1.04-1.82]. Women who reported health facility delivery were more likely to report complete thermal care [aOR: 3.63; 95% CI: 2.21-5.95] and newborn vaccination [aOR: 1.84; 95% CI: 1.23-2.75], but not complete cord care. Having the first baby was associated with complete thermal care [aOR: 2.00; 95% CI: 1.24-3.23].

**Conclusion:**

Results confirm suboptimal newborn care practices in Masindi. Despite being established policy, adherence to four or more ANC consultations was not associated with complete cord care, complete thermal care or complete newborn vaccination. This finding has important implications for the implementation of focused ANC to improve newborn care practices. Future ANC interventions should focus on addressing application of substance on the cord and early bathing of the baby during the immediate neonatal period.

## Background

Neonatal mortality has remained resistant to change [1,2]. Most causes of neonatal death are preventable and relate to cord care to decrease sepsis, temperature control and initiation of early breastfeeding which has the additional benefit of controlling hypothermia [3]. Good assisted deliveries can decrease delivery complications, stillbirth and early neonatal deaths [4]. Initiation of BCG and polio vaccination within the first four weeks after birth [5-7] can enhance neonatal survival.

One of the instruments to obtain reduction in neonatal mortality is Antenatal Care (ANC), where we now agree that the risk identification approach [8-10] is not the important reason. Rather, the preparation of pregnant women to become knowledgeable mothers [11] hence limiting the number of visits to four (at four, six, eight and nine months) [12] if no complications are anticipated [13,14]. ANC delivery should have as an important objective to inform and prepare women to care correctly for their newborn. Two mutually reinforcing interventions should be packaged and offered to pregnant women during ANC consultations. First, the more technical intervention which includes history taking, clinical examinations, laboratory investigations, treatment and assessment for referral [5]. Second, health education and counselling which entails dialogue that creates an interface between medical conditions and socially relevant issues regarding ANC such as promotion of healthy lifestyles among pregnant women, birth plans including preparation for unexpected events and preparation for parenting especially care for the newborn [15]. Throughout the ANC period information about physical and psychological preparation for the newborn is offered to pregnant women [6]. This process of communication is expected to progressively make the women better mothers.

Evidence in Asia indicates that information on newborn care practices offered to pregnant women and their families during ANC clinics leads to improved care practices (complete cord care, complete thermal care and initiation of breastfeeding within one hour) [16,17]. In order to achieve similar results, focused ANC whereby pregnant women are encouraged to make at least four ANC consultations, has been widely promoted in Uganda.

In Uganda, the coverage of focused ANC services has been scaled up to all tiers of the healthcare system with the aim of increasing awareness about birth preparedness, promoting supervised delivery and improving newborn care practices. Today, about 48% of pregnant women make four ANC consultations or more while 57% seek delivery assistance from a health facility [18,19]. However, recent studies conducted in Uganda have reported sub-optimal newborn care practices [20-23] and stagnated neonatal mortality rates [24]. Moreover, little is known about the relationship between newborn care practices and attending four ANC consultations.

This study specifically assessed newborn care practices with regards to thermal care, cord care and newborn vaccination status among lactating women in Masindi district in Uganda and determined its relationship with adherence to four or more ANC consultations.

## Methods

### Study design and setting

In a cross sectional design, lactating women with babies aged less than five months in Masindi and Kiryandongo districts were visited in their homes from October to December 2011. Similar studies conducted in Uganda have used age limits of less than five months [20]. At the time of conceptualizing this study, Masindi and Kiryandongo districts had one administrative unit, Masindi district and split to form two districts three months prior to data collection. We will therefore describe them assuming the original administrative unit by June 2011, hence Masindi district. Cluster survey was preferred to allow for generalizability to the entire district. Parishes were considered the sampling unit for this study. A parish in Uganda is a geographical area occupied by about 10,000 – 20,000 inhabitants.

Masindi district is located about 214 kilometres northwest of the capital city Kampala. The district population is 603,000 and about half (49.1%) are female. The population is predominantly rural with about 5.4% being urban. Administratively, Masindi district is divided into three counties (Buruli, Kibanda and Bujenje), eight sub counties and 41 parishes.

In Uganda, health care delivery in the public sector is decentralized to the district level. There are four tiers of care at the district level: general hospital, health centres at the county-level (health centre of level IV), sub county level (health centre of level III) and parish level (health centre of level II). The village health teams (an informal structure) based at the community provide a linkage between the community and the health centre of level II [25,26]. All health centres are mandated to offer ANC [25,27]. As a minimum requirement, each parish should be served by a health centre of level II.

In Masindi district there are two general Hospitals and one sub district hospital (Masindi, Kiryandongo and Bwijanga respectively), 16 health centres of level III and 26 health centres of level II. Health centre of level II offers ambulatory care services and treatment for mild illnesses. Health centre level III provides ambulatory care, in-patient and laboratory services. Hospitals are referral centres for the district health system. About 90% (37/41) of the parishes in Masindi district have a functional health centre as recommended by the Ministry of Health in Uganda and 80% of the population live within a five-kilometre walking distance to the nearest health centre [26]. In this region, routine data from the Health Management Information System indicates that 89.4% of pregnant women make at least one ANC consultation [28].

### Sample size and data collection procedures

The sample size was estimated using a standard formula for cluster surveys [29]. The prevalence of good cord care practices in the region was estimated to be 42% (based on similar studies conducted in eastern Uganda) [20], design effect of 2 and sample error at 5%. This yielded 18.7 clusters with a minimum sample size of 748. Adjusted for 10% none response, 823 participants from 21 clusters needed to be recruited.

List of parishes (clusters) were obtained from the planning department in Masindi district and 21 of them were randomly selected using computer-generated random numbers. At the parish level, local council leaders guided research assistants to households with lactating women having children less than five months old because we did not have a documented list of women of reproductive age nor did we have a list of women who had delivered. Structured interviews were held with consenting women in their homes. Where a household had two or more lactating women, only one was randomly enrolled. Data collected included socio-demographic characteristics, previous obstetric history and history of last pregnancy, immediate newborn care practices and immediate postnatal care-seeking practices in case of neonatal illness (see Additional file 1). Three primary outcome variables were considered of interest- cord care, thermal care and newborn vaccination status. None of the eligible mothers contacted declined participation.

Before data collection for the main survey, the questionnaire was pre-tested in the neighbouring Hoima district. Pre-test data were collected from 30 women in three parishes.

### Data analysis

Each questionnaire was checked for completeness before entry into Epidata software version 3.02. Double data entry was carried out and the two data sets were validated in the same software to eliminate entry errors. Analysis was carried out in STATA version 10.0. Socio-demographic characteristics, reproductive health indicators and selected newborn care practices were displayed using descriptive statistics. The number of ANC consultations during the last pregnancy was considered the exposure variable of interest and was coded as *sufficient ANC attendance = 1* defined as having made four ANC consultations or more and *insufficient ANC attendance = 0* defined as 1 to 3 antenatal consultations. Composite variables were generated from primary responses using the *egen* command in stata to constitute our primary outcome variables of interest. Outcome variables included *complete cord care* (defined as - used clean instrument to tie the cord, used clean instrument to cut the cord and applied salt water/nothing on the cord), *complete thermal care* (defined as - dried the newborn before the placenta or immediately after the placenta was delivered, wrapped the baby immediately the placenta was delivered and delayed bathing of the baby 24 hours or later after delivery) and *complete newborn vaccination* (a newborn baby who has received both tuberculosis and Polio zero vaccines routinely administered at birth). For purposes of our analysis we considered as *complete cord care* if any three of the five possible responses were correct, *complete thermal care* if any three of the four responses were correct and *complete newborn vaccination* if any one of the four possible outcomes were correct.

Independent variables considered during analysis were age (grouped as adolescents 15-19 years or adults 20-32 years), parity (having the first baby or having two or more babies), and marital status (living with spouse or living alone), source of income (regular or irregular source of income), level of education (attained no education/primary level only or secondary/and higher education level) and place of delivery (in health facility or delivered outside of health facility). The category ‘no education’ were grouped together with ‘primary education’ because we did not consider any difference between the two groups [30].

The chi-square test was used at the bivariate level of analysis. Independent variables with corresponding *p*-values of 0.4 or less were fitted into a logistic regression model. Number of antenatal care consultations was purposively maintained into the multi-variable model for each outcome variable of interest even when their corresponding *p*-value was greater than the significant levels because this was our primary exposure of interest. The *svyset* stata command was used to take care of the cluster effect of our data collection technique whereby people from the same geographical location tend to provide similar responses.

### Ethical consideration

A written consent was secured from all mothers before participation and confidentiality was guaranteed. This study was approved by the Higher Degrees Research and Ethics Committee of the School of Public Health, Makerere University and Uganda National Council for Science and Technology (Ref: HS 1145).

## Results

Nine hundred and twenty-eight lactating women were interviewed. Their mean age was 25.5 years [SD = 6.3: [95% CI: 25.1-25.9]. Nearly a fifth 175(18.9%) were women between 14 and 19 years old and one fifth 187(20.2%) were having their first baby. Majority 831(89.6%) lived together with their spouse, 608(65.5%) practiced subsistence farming. A quarter (235/928) of the women reported having lost at least one child in the years preceding the current baby. One in every four deaths (57/235) occurred in the first 28 days most of them early and were still births, while 57 percent of the deaths occurred in a health facility.

Two in five 42.2% (392/928) made four ANC consultations or more and a similar proportion 42.7% (396/928), reported giving birth in a health facility. Other characteristics of the mothers interviewed are presented in Table 1.

**Table 1:** Socio-demographic characteristics of mothers with children under five months (n = 928)

**Variable**	**n (%)**
**Age group**	
14-19 years	175 (18.9)
20-45 years	753 (81.1)
Mean age (Standard Deviation)	25.5 [6.3]
**Parity**	
One child	187 (20.2)
Two children	170 (18.3)
Three children	139 (15.0)
Four children	117 (12.6)
Five children	112 (12.1)
More than five children	203 (21.9)
**Education level of mother**	
Non	186 (20.0)
Primary	590 (63.6)
Secondary	143 (15.4)
Tertiary	9 (1.0)
**Marital status**	
Married/living with partner	831 (89.6)
Single	65 (7.0)
Separated	27 (2.9)
Widowed	5 (0.5)
**Source of income**	
Subsistence farming	197 (22.2)
Commercial farming	608 (65.5)
Self employed	109 (11.8)
Salaried job	14 (1.5)
**Women reporting death of at least a child**	
None	693 (74.7)
One	141 (15.2)
Two	63 (6.8)
Three	23 (2.4)
More than three	8 (0.9)
**Time of death of babies(**n = 235**)**	
Stillbirths	36 (15.3)
1-7 days after birth	13 (5.6)
7-28 days	8 (3.4)
More than 28 days after	178 (75.7)
**Where the baby’s death occurred from(**n = 235**)**	
Health facility	135 (57.4)
Home	100 (42.6)
**Number of ANC visits made**	
None	27 (2.9)
One	66 (7.1)
Two	146 (15.7)
Three	297 (32.0)
Four	252 (27.2)
Five or more	140 (15.1)
**Sufficient ANC visits**	
None	27 (2.9)
1-3 visits	509 (54.9)
> = 4 visits	392 (42.2)
**Place of delivery**	
Home	532 (57.3)
Health facility	396 (42.7)

### Newborn care practices

We examined newborn care practices among lactating women (Table 2) with a specific focus on cord care, thermal care and newborn vaccination status. Three in four women (702/928) used clean material for tying the cord, 92.2% (856/928) used clean instruments for cutting the cord, while 28.4% (264/928) applied salty water/nothing on the cord. About three out of five (542/928) dried their baby before/soon after the placenta was delivered, half (451/928) wrapped the baby before the placenta was delivered and 12.1% (112/928) reported delayed bathing of their baby by 24 hours or more after birth. By the time of the interview 84.3% and 64.8% of the babies had received BCG and Polio 0 vaccines respectively. The desired newborn care practices were assessed to be 23.7% (220/928) for complete cord care, 6.1% (57/928) for complete thermal care and 65.8 %( 611/928) for complete newborn vaccination status.

**Table 2:** Newborn care practices (n = 928)

**Variable**	**n (%)**
**Cord Care**	
**Material used for tying umbilical cord**	
Clean thread	702 (75.7)
Unclean household cloth	226 (24.4)
**Instrument used for cutting umbilical cord**	
New razor blade	534 (57.5)
Sterile instrument	322 (34.7)
Old razorblade/household instruments	72 (7.8)
**Application of substance on umbilical cord**	
Baby powder	576 (62.1)
Animal waste	5 (0.5)
Soot Powder	26 (2.8)
Herbs	57 (6.1)
Salt water/Spirit or alcohol	65 (7.0)
Nothing	199 (21.4)
**Cord Care**	
Incomplete cord care	**708 (76.3)**
Complete cord care	**220 (23.7)**
**Thermal care**	
**Warmth for the new-born/drying of the baby**	
Before the placenta is delivered	336 (36.2)
After the placenta is delivered	189 (20.4)
After the delivery is complete	17 (1.8)
Baby not dried just wrapped	386 (41.6)
**Warmth for the new-born/wrapping of the baby**	
Before the placenta is delivered	451 (48.6)
After the placenta is delivered	447 (48.2)
Long after the delivery has been concluded	30 (3.2)
**Warmth for the new-born/Timing of first bathing**	
Immediately after delivery of the baby	349 (37.6)
Within 6 hrs. of delivery	213 (23.0)
7-23 hrs. after delivery	254 (27.4)
Second day or later	112 (12.1)
**Thermal care**	
Incomplete thermal care	**871 (93.9)**
Complete thermal care	**57 (6.1)**
**Immunisation status**	
**Immunisation**	
Received BCG	782 (84.3)
Did not receive BCG	146 (15.7)
Received Polio 0	601 (64.8)
Did not receive Polio 0	327 (35.2)
**Incomplete newborn vaccination**	**317 (34.2)**
**Complete Newborn vaccination**	**611 (65.8)**

### Cord care

Results in the bivariate analysis for complete cord care (Table 3) indicate that education level [OR = 2.89: *p* < 0.001] and place of delivery [OR = 1.88: *p* = 0.032] were positively associated with complete cord care. Implying that having attended higher level of education and delivered in the health facility was significantly associated with providing complete cord care. Source of income [OR = 0.48: *p* = 0.005] was negatively associated with complete cord care.

**Table 3:** Complete cord care and demographic characteristics

**Variable**	**Incomplete cord care n (%)**	**Complete cord care n (%)**	**UOR [95% CI]**	***p*-value**	**aOR [95% CI]**	***p*-value**
Sufficient ANC**						
1-3 visits	389 (76.4)	120 (23.6)	1.0			
> = 4 visits	298 (76.0)	94 (24.0)	1.02 [0.70-1.95]	0.902	0.96 [0.65-1.41]	0.837
Age**						
14-19 yrs.	139 (79.4)	36 (20.6)	1.0			
20-42 yrs.	569 (75.6)	184 (24.4)	1.25 [0.80-1.95]	0.310	1.22 [0.82-1.83]	0.313
**Marital status**						
Not living with spouse	76 (78.4)	21 (21.7)	1.0			
Living with spouse	632 (76.1)	199 (24.0)	1.14 [0.74-1.76]	0.536		
Education**						
Non or primary	620 (79.9)	156 (20.1)	1.0			
Secondary plus	88 (57.9)	64 (42.1)	2.89 [1.72-4.45]	0.000	2.72 [1.63-4.54]	0.001*
**Parity**						
Two or more	566 (76.4)	175 (23.6)	1.0			
First child	142 (75.9)	45 (24.1)	1.02 [0.70-1.49]	0.906		
Place of delivery**						
Home	432 (81.2)	100 (18.8)	1.0			
Health facility	276 (69.7)	120 (30.3)	1.88 [1.06-3.32]	0.032	1.54 [0.84-2.81]	0.150
Source of income**						
Subsistence farming	128 (65.0)	69 (35.0)	1.0			
Commercial farming/regular income	580 (79.3)	151 (20.7)	0.48 [0.30-0.78]	0.005	0.54 [0.32-0.90]	0.021*

**For initial inclusion in multivariable model; *p-is less than 0.05; UOR = unadjusted odds ratio; aOR = adjusted odds ration; (95% CI) Confidence Interval at 95%.

Independent variables included into a multivariable level analysis for complete cord care were sufficient ANC visits, age of the mother, education level, and place of delivery and woman’s main source of income. In the adjusted analysis (Table 3) women who practiced commercial farming [(46%): *p* = 0.021] were less likely to report complete cord care. While women that had attained at least secondary education [(172%): *p* = 0.001] were more likely to report complete cord care, adjusted for age and place of delivery. Sufficient ANC visit was not significantly associated with reporting complete cord care (aOR = 0.96: *p* = 0.837).

### Thermal care

Preliminary analysis showed that women who were having their first baby (uOR = 2.28: *p* = 0.001) and women who delivered in the health facility (uOR = 3.72: *p* < 0.001) were more likely to report complete thermal care. For further analysis the variables age, education, parity, delivery place and occupation were included in to the multivariable model for complete thermal care (Table 4). Sufficient ANC visit was maintained as the exposure variable of interest. Mothers who were having their first child [(100%): *p* = 0.007], had a health facility delivery [(263%): *p* < 0.001] were more likely to report complete thermal care adjusting for age, education level and main source of income; implying that having the first child and delivering in the health facility were positively associated with reporting complete thermal care. Sufficient ANC consultation was not associated with reporting complete thermal care (aOR = 1.07:*p* = 0.858).

**Table 4:** Complete thermal care and demographic characteristics

**Variable**	**Incomplete thermal care n (%)**	**Complete thermal care n (%)**	**UOR [95% CI]**	***p*-value**	**aOR [95% CI]**	***p-*** **value**
Sufficient ANC**						
1-3 visits	481 (94.5)	28 (5.5)	1.0			
> = 4 visits	364 (92.9)	28 (7.1)	1.32 [0.61-2.85]	0.458	1.07 [0.50-2.30]	0.858
Age**						
14-19 yrs.	161 (92.0)	14 (8.0)	1.0			
20-42 yrs.	710 (94.3)	43 (5.7)	0.70 [0.43-1.12]	0.130	1.13 [0.59-2.17]	0.696
**Marital status**						
Not living with spouse	90 (92.8)	7 (7.2)	1.0			
Living with spouse	781 (94.0)	50 (6.0)	0.82 [0.29-2.30]	0.697		
Education**						
None/primary	733 (94.5)	43 (5.5)	1.0			
Secondary plus	138 (90.8)	14 (9.2)	1.73 [0.89-3.32]	0.098	1.08 [0.45-2.57]	0.857
Parity**						
Two or more	704 (95.0)	37 (5.0)	1.0			
Having First child	167 (89.3)	20 (10.7)	2.28 [1.50-3.47]	0.001	2.00 [1.24-3.23]	0.007*
Place of delivery**						
Home	516 (97.0)	16 (3.0)	1.0			
Health facility	355 (89.7)	41 (10.4)	3.72 [2.34-5.93]	0.000	3.63 [2.21-5.95]	0.000*
Occupation**						
Subsistence farming	182 (92.4)	15 (7.6)	1.0			
Commercial farming/regular income	689 (94.3)	43 (5.8)	0.74[0.41-1.32]	0.291	0.95[0.53-1.71]	0.869

**For initial inclusion in multivariable model; *p-less than 0.05 UOR = unadjusted odds ratio; aOR = adjusted odds ration; (95% CI) Confidence Interval at 95%.

### Vaccination status

In relation to newborn vaccination status (Table 5), sufficient ANC visits (uOR = 1.50:*p* = 0.024), having attained secondary education or higher (uOR = 1.75: *p* = 0.001) and reporting delivery at health facility (uOR = 2.13:*p* < 0.001) were significantly associated with reporting complete newborn vaccination status. At the multivariable level, having attained secondary education or higher [37%: *p* = 0.029] and women who delivered in the health facility [84%: *p* < 0.001] were significantly associated with reporting complete newborn vaccination status adjusted for age and parity. The level of statistical significance was not sustained for making sufficient ANC visits when adjusted for age and parity (aOR = 1.34: *p* = 0.133).

**Table 5:** Complete newborn vaccination and demographic characteristics

**Variable**	**Incomplete newborn vaccination n (%)**	**Complete newborn vaccination n (%)**	**UOR [95% CI]**	***p*-value**	**aOR [95% CI]**	***p*-value**
Sufficient ANC**						
1-3 visits	191 (37.5)	318 (62.5)	1.0			
> = 4 visits	112 (28.6)	280 (71.4)	1.50 [1.06-2.13]	0.024	1.34 [0.91-1.99]	0.133
Age**						
14-19 yrs.	65 (37.1)	110 (62.9)	1.0			
20-42 yrs.	252 (33.5)	501 (66.5)	1.17 [0.91-1.52]	0.202	1.25 [0.94-1.66]	0.115
**Marital status**						
Not living with spouse	31 (32.0)	66 (68.0)	1.0			
Living with spouse	286 (34.4)	545 (65.6)	0.90 [0.50-1.60]	0.695		
Education**						
None/ Primary	280 (36.1)	496 (63.9)	1.0			
Secondary plus	37 (24.3)	115 (75.7)	1.75 [1.29-2.38]	0.001	1.37 [1.04-1.82]	0.029*
Parity**						
Two or more	256 (35.0)	482 (65.1)	1.0			
Having First child	58 (31.0)	129 (69.0)	1.20 [0.76-1.87]	0.417	1.37 [0.72-2.61]	0.323
Place of delivery**						
Home	219 (41.2)	313 (58.8)	1.0			
Health facility	98 (24.8)	298 (75.2)	2.13 [1.47-3.08]	0.000	1.84 [1.23-2.75]	0.005*
**Occupation**						
Subsistence farming	71 (36.0)	126 (64.0)	1.0			
Commercial farming/ Regular income	246 (33.7)	485 (66.4)	1.11 [0.72-1.72]	0.622		

**For initial inclusion in logistic model; * *p*-is less than 0.05; UOR = unadjusted odds ratio; aOR = adjusted odds ration; (95% CI) Confidence Interval at 95%.

## Discussion

Our data confirm sub-optimal newborn care practices for cord, thermal and vaccination status of the newborn. Thermal care ranked least practiced followed by cord care. Newborn vaccination was relatively better practiced. Women who reported last delivery being at a health facility were more likely to report complete thermal care and complete newborn vaccination status. But health facility delivery was not a good predictor of complete cord care. Studies conducted in Bangladesh suggest increasing the number of skilled birth attendants as an effective strategy to increase safe delivery practices [31]. Indeed there is a general consensus that access to skilled attendants at delivery has multiple benefits for the mother-baby pair [32]. Studies conducted elsewhere show that women who delivered in health facilities were more likely to report the practice of recommended newborn care [31,33]. Community interventions may help to reinforce recommended cord care practices initiated from the health facility to mitigate the problem of incomplete cord care identified in our study.

### Cord care

From our results, the main determinant of complete cord care was application of potentially infectious substance on the cord. Majority of such substances are applied in the homes after discharge from a health facility and away from the supervision of a qualified health worker. Our results are comparable to Waiswa and others in eastern Uganda, where only 38% of the women [20] and in Bangladesh where 42.8% of the women were considered to have practised good cord care [31]. Elsewhere, application of substance on the cord was reported to face the greatest resistance to change [34,35]. Future cord care interventions should explore alternative substances that could be safely applied to the cord [36]. Women who had attained secondary education or more were more likely to report complete cord care. Similar studies conducted in Bangladesh found that secondary education was significantly associated with recommended newborn care practices including good cord care [33]. Women that were engaged in commercial farming or had a regular income, though counter-intuitive, were less likely to report complete cord care. It is possible that women who are engaged in commercial farming and earning regular incomes are more often absent from their homes and thus less available for child care.

### Thermal care

Less than one in ten of the mothers were judged to practice recommended thermal care. Our results are comparable to another study conducted in Bangladesh where 5.1% were judged to have complete thermal care [31]. In our study, early bathing of the baby within the first 24 hours following birth was the main driver for the low thermal care practices. In eastern Uganda, a similar study found that nearly all mothers had bathed their babies within the first 24 hours after delivery [20]. A set of interlinked procedures referred to as the “warm chain” suggest the provision of warmth to the newborn from the time of birth and throughout the neonatal period [7]. Delayed bathing is one component of the warm chain. A controlled study conducted in Uganda demonstrated that early bathing significantly lowered neonatal body temperatures even when bathed with warm water and later applied skin-to-skin warming for the baby [23]. Moreover, in one hospital set up in Uganda 80% of the babies were recorded to have hypothermia within the first two hours following delivery [22]. Unfortunately, the practice of bathing the baby immediately after delivery [37] and delayed drying and wrapping [38] have also been encouraged by health workers based at the hospital. Another study that explored thermal care offered to newborns in Ghana suggests that interventions should be based on understanding of current behaviours and beliefs and must focus on messages and approaches in order to overcome barriers to behaviour change [39]. Indeed one study conducted in southern Uganda showed that most of the newborn care practices like thermal care are readily acceptable [21] by recently delivered women although other aspects of care like early bathing and application of substance to the cord are still resisted as they contravene societal cultural norms [40].

### Newborn vaccination status

Newborn vaccination compared with cord and thermal care was better practiced. This may be in part due to the higher attention health workers accord to vaccination during prenatal care and immediate postnatal period [41]. Our results demonstrate that complete newborn vaccination status was significantly related to having health facility delivery and mother having secondary education or higher. This is in agreement with studies conducted elsewhere in Uganda [42,43] and in Kenya [44]. These studies further showed that children with several siblings were more likely to have untimely vaccinations. Our study showed positive relationship between women having their first baby and reporting complete newborn vaccination although our results were not statistically significant. This difference could have been as a result of studying different age groups. Their study enrolled children 10-23 months old while our study considered children less than five months old.

### Policy implications

Consistently, adherence to four or more ANC visits did not show statistically significant relations with newborn care practices implying that ANC is not delivering on the desired newborn care practices. We expected that women who adhered to recommended four ANC consultations would report better newborn care practices compared to those non-adherent mothers. This finding raises important questions about the organization and offer of ANC education already highlighted in a separate study conducted in the same region [41]. It further has implications for implementing the policy guidelines suggested by WHO for a comprehensive mother-newborn care package [45,46], the policy on focused ANC and subsequently the practices of newborn care.

WHO recommends at least four ANC consultations during pregnancy with a specific focus for every visit [5] hence focused ANC [47]. The focus of these visits are a comprehensive package that includes five main elements: **1**) recognition and management of pregnancy related complications; **2**) recognition and treatment of concurrent conditions; **3**) screening for conditions and diseases; **4**) preventive measures like administration of tetanus toxoid and intermittent presumptive treatment of malaria; **5**) advice and support to the woman and her family for healthy home behaviours and birth preparation plans, self care, recognition of danger signs, early care seeking, emotional and physical preparation for birth and care for the baby.

Clearly, the first four packages are clinically oriented requiring the input from skilled personnel like a midwife, but the fifth element which pertains to advice and support may not necessarily require such high level skills. We hypothesize that health workers in Masindi are more inclined to offer clinically oriented services at the expense of less technical services like health education [39,48]. Even when some women made adequate contact with the health care system during pregnancy they remained inadequately prepared for newborn care also demonstrated in a separate paper [41]. Health workers could inadvertently ignore components of ANC that relate to advice and support to the mother and their family including immediate newborn care. For example, a study in western Kenya showed that provision of palpation and vaccination during antenatal care were over 90% while those who received health education were only 14.4% [49]. In eastern Uganda, mothers interviewed could not recall any information given to them on pregnancy or newborn care [39]. In a multi-country study [50] in Africa, less than 50% of the women reported they did not receive any educational information regarding danger signs in pregnancy leading to the delays in seeking for care in case of complications. Contrary to our findings, women who completed three or more ANC visits in Bangladesh were more likely to report having practiced recommended newborn care [31]. Also in Bangladesh a study conducted among the ultra-poor populations suggests that specific subclass of people required a tailored educational intervention in order to attain a behaviour change in the uptake of pre-and post-natal services [51]. It is possible that health workers in Masindi do not consider these unique aspects of the women while offering information regarding newborn care.

### Strengths and limitations

Composite variables were derived from the different aspects of newborn care practices therefore introducing a measure of consistency in the recommended care practices. Individual care practices considered separately would somehow be misleading; for example over 90% of mothers reported using clean instruments for cutting the cord and yet only one third did not apply harmful substances on the umbilical cord. We hasten to add that while a composite measure gives more accurate measures of newborn care practices, most of these practices are beyond control of the mother alone. For example, the person who offers delivery care will tie the cord and cut the cord without necessarily any input from the mother. In the same way drying, wrapping and bathing of the baby remains under the control of the person assisting her in the delivery or the relatives who will attend to her soon after delivery. This may in part explain why delivering in a health facility was a predictor of thermal care and newborn vaccination. Nevertheless, a mother is likely to have a stronger influence over the newborn care interventions at the time of delivery if she has been adequately prepared with the correct information during ANC consultations [52].

In Uganda the Expanded Program on Immunization recommends the offer of vaccination for BCG and Polio 0 at birth although WHO provides a timeframe between zero to 8-weeks for BCG and zero-4 weeks in case of Polio 0. Our study could have under-estimated the vaccination status for newborns given that some of the babies were still within the age range of zero and 8 weeks. However, we think that mothers who had not had their babies vaccinated by the time of this study were also less likely to have a timely vaccination [44,53].

Creation of a binary outcome for ANC consultations means grouping together one and three ANC consultation, yet making three ANC consultations are not necessarily equivalent to making one visit. We attempted to analyze separately for one, two, three, four or more consultations but could not establish any gradient (results not shown). The data collected were self-reported some women could have offered incorrect information due to recall bias. Direct observations could have mitigated this limitation in our study.

## Conclusion and suggestions

Our results reaffirm the suboptimal newborn care practices. Application of potentially harmful substance on the cord and early bathing of the newborn are some of the drivers of inappropriate newborn care practices. Contrary to recommendations by the World Health Organization, this study could not demonstrate that adherence to four or more ANC consultations is an independent predictor of complete cord, complete thermal care or complete newborn vaccination. This has important implications for the implementation of the policy of focused ANC.

These findings ought to be interpreted with caution and within the context of the Ugandan local health system. We suggest further enquiry into the content and scope of prenatal services offered to pregnant women during ANC consultations in Masindi district. Application of substance and early bathing of the baby should receive priority attention during the immediate neonatal period (0-72 hours).

## Abbreviations

ANC: Antenatal care; BCG: Bacillus Calmette–Guérin; WHO: World Health Organisation.

## Competing interests

The authors declare that there is no competing interests.

## Authors’ contributions

RMA, BC, CGO and PK conceptualized and designed the study. RMA entered the data; RMA, SK and PK analyzed the data. RMA, SK and PK wrote the manuscript. All authors read and approved the approved the final manuscript.

## Pre-publication history

The pre-publication history for this paper can be accessed here:

http://www.biomedcentral.com/1471-2393/14/114/prepub

## Supplementary Material

Additional file 1 Data collection tool for lactating women. Click here for file
