# Supplementary material for: Is antenatal care preparing mothers to care for their newborns? A community-based cross-sectional study among lactating women in Masindi, Uganda
Source: BMC Pregnancy Childbirth. 2014 Mar 25;14:114. doi: 10.1186/1471-2393-14-114 (PMC3987096; doi:10.1186/1471-2393-14-114)
Supplement: Additional file 1 — Data collection tool for lactating women. [file 1471-2393-14-114-S1.docx]

**Is antenatal care preparing mothers to care for their newborns? A community-based cross-sectional study among lactating women in Masindi District, western Uganda**

Data collection tool for lactating women

**A: Socio-demographic characteristics**

1. Code number..........................................................................................
2. Name of household Head........................................................................
3. Parish……………………………………………………………………
4. Age of Respondent in completed years (lactating woman)....................
5. Parity of respondent 1, 2, 3, 4, 5, over 5
6. Highest education level attained:
7. Non
8. Primary;
9. Secondary;
10. High school;
11. Tertiary;
12. Marital status:
13. Married
14. Single
15. Separated
16. Divorced
17. Widowed
18. What do you do for a living:
19. Nothing
20. Housewife
21. Farming/gardening
22. Selling in the market/petty trade
23. Business woman
24. Salaried
25. Did you attend any ANC during the last pregnancy:
26. Yes, 2. No (***skip to no. 11***)
27. Number of ANC visits made with the last pregnancy:
28. 0,
29. 1,
30. 2,
31. 3,
32. 4
33. More than 4
34. Where did this last delivery take place:

**1)** Home;

**2)** Health facility;

**3)** On the way to health Facility

1. How old is the baby now? Age in completed weeks
2. 0-1 week **2)** 2-4 weeks **3)** 5-8 weeks **4)** 9-13 weeks
3. Number of living children: 1, 2, 3, 4, 5, 6, more than 6
4. Number of deceased children: 0,1, 2, 3, 4, 5, 6, more than 6

(*if deceased babies is* ***0*** *then skip to section* ***B***)

1. At what ages did the deceased children die?

**a) First death,**

1) Died as a still birth

2) Within 24 hours

3) After 24 hours but within 72 hours

4) After 72 hours but within 7 days

5) After 7 days but within 28 day

6) After 28 days

**b) Second death,**

**1**) Died as a still birth

**2**) Within 24 hours

**3**) After 24 hours but within 72 hours

**4**) After 72 hours but within 7 days

**5**) After 7 days but within 28 day

**6**) After 28 Days

**c) Third death,**

**1**) Died as a still birth

**2**) Within 24 hours

**3**) After 24 hours but within 72 hours

**4**) After 72 hours but within 7 days

**5**) After 7 days but within 28 day

**6**) After 28 Days

13. Indicate where the deceased babies were born from:

**1)** At home

**2)** At health facility

**3)** On the way to Health facility

**B: Data on the last pregnancy**

1. During your last pregnancy is there any food that was forbidden for you to eat?

1=Yes

2=No

1. If YES why?

1) will make the baby grow too big

2) will cause the baby to come out;

3) other, Specify...................

1. During this pregnancy were you advised not to take part in certain activities?

1=Yes

2=No

1. If YES, please *(circle all that apply)*:

1) Not going to the well to fetch water

2) Not using a pit latrine

3) Not engaging in sexual intercourse

4) Other, please Specify...................

1. Did you make any preparation for this delivery?

1=Yes

2=No

1. If YES, what specific preparations did you make? *(Circle all that apply)*

**1**) Secured warm dresses and other items for the baby like basin,

**2**) mama kit, mackintosh,

**3**) saved some money,

**4**) decided where to deliver from,

**5**) selected a caretaker for the remaining children while I am away ,

**6**) other, please specify

**C: Information on Delivery**

1. Mode of delivery of the current baby:

**1**) Spontaneous Vaginal delivery;

**2**) C/S

**3**) vacuum;

4) Forceps

1. Place of delivery:

**1)** health centre,

**2)** hospital

**3)** home

**4**) on the way to Health Facility

**5)** At the TBAs home

1. Who attended to you during delivery

**1**) By trained health worker

**2**) A close relative

**3**) By TBA

**4)** Unattended to

1. Timing of labour-in your view was this labour:

**1)** Normal

**2)** Longer than usual

**3)** Too long

**4)** Too short

**5)** No idea

1. Did you develop any intra-partum complication: *(Circle all that apply)*
2. too long labour
3. bleeding
4. swelling of the feet
5. convulsions
6. ruptured uterus
7. obstructed labour
8. non

**D. Clean delivery**

1. What instrument was used for cutting the cord?

1. New razorblade
2. Sterilised instrument
3. Used/old razor blade
4. A knife
5. Other sharp objects used in the household
6. What material was used to tie the cord?
7. Clean thread was used
8. Application of spirit/alcohol
9. Thread from household cloth
10. Pieces of used cloth
11. What substance was applied on the cord?
12. Application of powder on the stump
13. Application of animal waste
14. Application of soot powder
15. Salt water
16. Herbal medicines
17. Nothing

**E. Warmth**

1. At what stage after delivery was the newborn dried up

1. Immediately before the placenta was delivered
2. Immediately after delivery of the placenta
3. After the mother was cleaned and wrapped
4. After the whole process of the delivery was completed
5. Not dried, just wrapped

2. At what stage was the newborn wrapped up in a warm cloth

1. Immediately before the placenta was delivered
2. Immediately after the placenta was delivered
3. Long after the delivery was competed

3. How soon after delivery was the first bath?

1. Immediately after delivery;
2. Not immediately but within 6 hours;
3. 7-23 hours after;
4. Second day after delivery or later

**F. Assessment of newborn**

1. Was there any need for Resuscitation of the newborn?

1=Yes

2=No? (If no then skip to no.3)

2. If YES, what method was used for resuscitation?

1. Hanged the legs up,
2. Sprinkled cold water,
3. Mouth-to-mouth resuscitation
4. Slap the baby
5. Oxygen
6. Nothing

3. How was the weight of the newborn assessed?

1. Using a weighing scale
2. Estimation by looking at the size
3. Not done

4.1. If baby was premature how was assessment of prematurity done? (*If the response is NO then skip to section* ***G)***

1. Size of the baby
2. Told by health practitioner
3. Weight of the baby
4. Gestation age of the pregnancy
5. Anthropometric measurements

4.2. If Anthropometric measurements which one?

1. Feet
2. Arm
3. Head
4. thigh

5. Was there any special care given to this premature newborn baby?

1=YES

2=NO (***if no then skip to section G***)

6. If YES in 5 above, What remedy was offered to the newborn? (***please tick as many options***)

1. Extra warmth
2. Extra feeds
3. Frequent feeds
4. Less frequent bathing
5. No bathing at all
6. Keeping the baby indoor only
7. Specific traditional performances

**G. Care-seeking practices**

1. Did you make any postnatal visits to the health facility

1=Yes

2=No (***if no then skip to Section H***)

1. How many postnatal visits so far have you made: 1,2,3,4
2. How early was the first visit
3. Within the first 24 hours after delivery
4. After 24 hours but before 72 hours
5. After 72 hours but before one week
6. After one week
7. After one month

**H. Care seeking**

Newborn immunization

1. Is the newborn already immunized for

1. Polio zero 1=Yes 2=No
2. BCG 1=Yes 2=No
3. Polio 1 1=Yes 2=No
4. DPT HEB+Hib (pentavalent) 1=Yes 2=No

2.Did this newborn fall ill in the last three months? 1=Yes 2=No)

(*if NO conclude the interview*)

3. If yes how did you detect the illness? *(Circle all that apply)*

1. Failure to feed
2. Excessive crying
3. Less activity
4. Warm to touch

4. What immediate actions did you take? *(Circle all that apply)*

1. Attention to VHT
2. To Health centre
3. Seek medication at home
4. Traditional healers
5. Went to private clinic
6. Did nothing

5. How soon after the onset of illness did you seek for care?

1. Within one hour
2. Within six hours
3. Within 24 hours
4. After 24 hours
5. What in your view was the cause of illness?
6. Evil spirit
7. Some evil neighbours or relatives
8. Bad air
9. The infected umbilical stump
10. Acquired from place of delivery
11. Acquired from mother
12. No idea

Thank you for your time
